# Phosphopantetheinyl transferase ClbA contributes to the virulence of avian pathogenic *Escherichia coli* in meningitis infection of mice

**DOI:** 10.1371/journal.pone.0269102

**Published:** 2022-07-28

**Authors:** Xia Meng, Yanfei Chen, Peili Wang, Pengpeng Xia, Jinqiu Wang, Mengping He, Chunhong Zhu, Heng Wang, Guoqiang Zhu

**Affiliations:** 1 Jiangsu Co-innovation Center for the Prevention and Control of Important Animal Infectious Diseases and Zoonoses, College of Veterinary Medicine, Yangzhou University, Yangzhou, Jiangsu, China; 2 Joint International Research Laboratory of Prevention and Control of Important Animal infectious Diseases and Zoonotic Diseases, Yangzhou University, Yangzhou, Jiangsu, China; 3 Joint International Research Laboratory of Agriculture and Agri-Product Safety, The Ministry of Education of China, Yangzhou, Jiangsu, China; 4 Department of Animal Husbandry and Veterinary Medicine, Beijing Agricultural Vocational College, Beijing, China; 5 Jiangsu Institute of Poultry Science, Yangzhou, Jiangsu, China; CINVESTAV-IPN, MEXICO

## Abstract

Avian pathogenic *Escherichia coli* (APEC), which has potential zoonotic risk, can cause severe systemic infections such as septicemia and meningitis in poultry. Colibactin is a hybrid non-ribosomal peptide/polyketide secondary metabolite produced by bacteria, which induces double-strand DNA breaks and chromosome instability in eukaryotic cells. ClbA is a 4’-phosphopantetheinyl transferase (PPTase) that is essential for colibactin and plays a role in siderophore synthesis. However, whether ClbA is associated with meningitis development in APEC is unclear. In this study, we abolished the *clbA* gene in the APEC XM strain, investigated the effect of *clbA* on colibactin synthesis and evaluated the pathogenic capacity of colibactin on meningitis development. Deletion of *clbA* reduced DNA damage to cells and hindered the normal synthesis of colibactin. Compared with the mice infected by wild-type APEC XM, the *clbA* deletion mutant infected mice had significant reduction in a series of characteristics associated with meningitis including clinical symptoms, bacterial loads of blood and brain, disruption of the blood brain barrier and the expression of inflammatory factors in the brain tissue. Complementation of ClbA recovered some APEC XM virulence. We conclude that ClbA is obligatory for the synthesis of colibactin and is responsible for the development of meningitis in mice infected by APEC.

## Introduction

Avian pathogenic *Escherichia coli* (APEC) is an extra-intestinal pathogenic *Escherichia coli* (ExPEC), which causes severe systemic infections such as meningitis and septicemia in poultry. The rate of isolation of meningitis-causing APEC from poultry is increasing, leading to an increasing proportion of neurological symptoms [1, 2]. APEC strains with serotypes O18 and O2 cause meningitis in newborn mice and ducks. They harbor many similarities with neonatal meningitis *E*. *coli* (NMEC) in genomic structure and toxic factors, such as *ibeA*, *ibeB*, and *gimB* [3–5]. Therefore, APEC is considered to share similar virulence gene contents and pathogenic ability with ExPEC and it has potential zoonotic risk.

Colibactin is a natural genotoxin that induces DNA double-strand breaks and damage in eukaryotic cells, leading to cell cycle arrest and megalocytosis [6, 7]. Colibactin is synthetized by a hybrid non-ribosomal peptide synthetase-polyketide synthase (NRPS-PKS) assembly line, which is encoded by a 54-kb genomic island (*pks* island) [6]. The *pks* island was initially identified in a NMEC strain IHE3034. It is also found in other *E*. *coli* strains such as ExPEC, commensal strains [6] and in *Klebsiella pneumoniae* [8]. *E*. *coli* harboring the *pks* island (*pks*^+^ *E*. *coli*) is over-represented in newborns with neonatal meningitis and sepsis, and it exacerbates development of systemic pathologies [9]. In addition, *pks*^+^ *E*. *coli* also causes a mutational signature in colorectal cancer [10] and promotes colon tumor growth [11]. The *pks* island consists of a total of 19 genes (*clbA* to *clbS*) [6]. ClbA is a 4’-phosphopantetheinyl transferase (PPTase) that is essential for biosynthesis of colibactin [6]. ClbA also contributes to siderophore biosynthesis. ClbA is important for maintaining the full virulence of a phylogroup B2 ExPEC strain in a mouse model of sepsis [12]. The deletion of *clbA* hindered *Klebsiella pneumoniae* hypervirulence in the meningitis development of BALB/c mice [13].

APEC XM (O2:K1), a B2 phylogenetic group *E*. *coli* strain used in this study, was isolated from the brains of ducks infected with meningitis and sepsis. This strain causes severe meningitis in 5-week-old mice, newborn SD rats and ducks [14, 15]. However, the meningitis-causing mechanism of APEC has not been clarified. The genome of APEC XM carries the *pks* island. The expressions of *pks* island genes such as *clbA* and *clbG* are induced when APEC XM infects mouse microvascular endothelial cell line (bEnd.3) [16]. The possible *clbA* contribution to meningitis development in APEC XM has not been reported. In this study, we deleted the *clbA* gene in APEC XM and evaluated a series of phenotypes associated with meningitis development in *clbA* deletion mutant in mouse meningitis infections.

## Materials and methods

### Bacterial strains, plasmids, and growth conditions

The bacterial strains and plasmids used in this study are listed in Table 1. The APEC XM strain (O2:K1) was donated by Dr. Chengping Lu, Nanjing Agricultural University. It was isolated from a duck brain with symptoms of septicemia and meningitis. The *clbA* deletion mutant and complemented mutant were derived from the APEC XM. All bacteria were grown aerobically on Luria-Bertani (LB) plates or in LB broth at 37°C with agitation (180 rpm), except for the mutants containing the temperature-sensitive plasmid pCP20 or pKD46, which was grown at 30°C. Strains harboring antibiotic resistance genes were cultured in LB containing ampicillin (Amp, 100 μg/mL) (Sangon Biotech, Shanghai, China) or chloramphenicol (Cm, 34 μg/mL) (Sangon Biotech, Shanghai, China) when appropriate. Plasmids pKD3, pKD46 and pCP20 were used for the λ-Red mediated recombination system. pBR322 was used for the construction of the complemented mutant. To determine growth rates, bacteria were incubated at 37°C in LB broth for 24 h with continuous agitation (180 rpm). The number of live bacteria was measured at 1 h intervals by determining the optical density (OD) at 600 nm. The growth curve experiment was performed with three biological replicates.

**Table 1:** Bacterial strains and plasmids used in this study.

Strain or plasmid	Characteristic or function	Source and reference
APEC XM	Virulent strain of APEC	Donated by Dr. Chengping Lu
APEC XMΔ*clbA*	Deletion mutant of *clbA* with APEC XM background	This study
APEC XMΔ*clbA*/p*clbA*	APEC XM Δ*clbA* carrying the vector pBR-*clbA*, Amp^r^	This study
pKD46	Amp^r^, λ red recombinase expression	[17]
pKD3	Cm^r^; Cm cassette template	[17]
pCP20	Amp^r^, Cm^r^, Flp recombinase expression	[17]
pBR-*clbA*	Amp^r^, pBR322 carrying the entire *clbA* nucleotide sequence	This study

### Construction of the clbA deletion mutant and the complemented mutant

All primers used for mutant construction are listed in S1 Table. The *clbA* deletion mutant was constructed using the λ-Red mediated recombination system as previously described [17, 18]. Briefly, primer pairs ClbA-F and ClbA-R were used to amplify the chloramphenicol resistance (Cm^r^) cassette from plasmid pKD3, including 50-bp homology extensions from the 5’ and 3’ of the *clbA* gene sequence. The polymerase chain reaction (PCR) product was purified and introduced into plasmid pKD46-containing APEC XM to construct the Cm^r^ recombinant bacteria, and then, the Cm cassette was removed using plasmid pCP20. The *clbA* complete deletion mutant APEC XM△*clbA* was confirmed by PCR screening using primers (ClbA-VF and ClbA-VR) and DNA sequencing (S1 Fig). For the generation of the complemented strain, the full-length *clbA* sequence was cloned into plasmid pBR322 using primer pair pBRclbA-F and pBRclbA-R. The recombined plasmid pBR-*clbA* was transformed into the *clbA* deletion mutant to generate the complemented mutant.

### Determination of the genotoxic effect induced by colibactin

The expression of γ-H2AX in bEnd.3 cells was determined to assess the genotoxicity induced by colibactin [6]. The bEnd.3 cells were infected with APEC XM, APEC XMΔ*clbA*, or APEC XMΔ*clbA*/p*clbA* for 4 h. Cells were washed three times with PBS and incubated in Dulbecco’s minimal Eagle medium (DMEM; Gibco, USA) with 10% fetal bovine serum (FBS; Gibco, USA) containing gentamicin (100 μg/mL). Then, the expression of γ-H2AX was detected immediately (at 0 h) or at 72 h post-incubation. The cells were washed three times and fixed with 4% paraformaldehyde for 20 min and then permeabilized with 0.1% Triton X-100 for 20 min and processed for immunofluorescence following a standard protocol [19] using the primary antibody (monoclonal rabbit anti phosphorylated H2AX, Cell Signaling Technology, MA, USA) and the secondary antibody (goat-anti-rabbit IgG (H+L) Alexa Fluor Plus 488, Thermo Fisher Scientific, CA, USA). Then, the cells were stained with 4’, 6-diamidino-2-phenylindole (DAPI, Beyotime Biotechnology, Shanghai, China). Finally, the coverslips were fixed using a fluorescence mounting medium. The GFP fluorescence was detected and photographed by a fluorescence microscope (Leica, Weztlar, Germany).

### Megalocytosis determination induced by colibactin

Mouse microvascular endothelial cell line bEnd.3 (American Type Culture Collection, ATCC CRL-2299) was used to determine the cytotoxic effect induced by colibactin on eukaryotic cells. The cells were cultured in DMEM with 10% heat-inactivated FBS at 37°C in an atmosphere of 5% CO_2_. Megalocytosis determination was performed as described previously [12]. Briefly, bacteria were cultured to log phase and washed with DMEM. The bEnd.3 cell cultures (about 75% confluence) were infected with a multiplicity of infection (MOI) of 100. At 4 h post-inoculation, cells were washed three times with PBS and incubated in DMEM with 10% FBS containing gentamicin (100 μg/mL) for 72 h. The cells were fixed with 4% paraformaldehyde for 20 min and then stained with 0.1% methylene blue for 20 min. The methylene blue was extracted with HCl, and the quantification of staining was measured at OD_600 nm_ using a microplate reader.

### Establishment of mouse meningitis model infected with APEC XM

The animal experiments followed the National Institute of Health guidelines for the ethical use of animals in China. All animal protocols were approved by the Animal Care and Ethics Committee of Yangzhou University and carried out in accordance with the approved guidelines. All research staffs have been trained in animal care and handling. Four-week-old Institute of Cancer Research (ICR) mice were provided by the Comparative Medicine Center of Yangzhou University (License number: SCXK (Su) 2017–0007). All mice had free access to food and water under a 12:12 h (L:D) photoperiod.

Bacteria were grown in LB to log phase, collected by centrifugation and diluted in PBS prior to inoculation into mice. Forty 4-week-old ICR mice were randomly separated into negative control group, APEC XM infection group, APEC XMΔ*clbA* group and APEC XMΔ*clbA*/p*clbA* group. Each group included ten mice (five males and five females). Each mouse was intraperitoneally injected with a dose of 10^7^ CFU bacteria in 100 μL normal saline, or with 100 μL sterile normal saline in control group. The status and clinical symptoms of mice were monitored per hour throughout the experiment. The health status of the mice was assessed by a clinical score method which was described previously (0, no apparent behavioral abnormality; 1, moderate lethargy; 2, severe lethargy; 3, unable to walk; 4, dead) [20]. When reaching clinical score 3, the mouse was sacrificed for ethical reasons. None of the animals died spontaneously. At 12 h post infection, 10 mice from each group were randomly chosen for Evans blue (EB) (Sigma, USA) permeability assay. All efforts were made to minimize suffering. Then the mice were sacrificed by manual cervical dislocation, which resulted in enthanasia within approximately 10 second. Once euthanasia was confirmed, blood, brains, spleen and lungs were immediately collected. None of mice died spontaneously before enthanasia.

### Determination of bacterial loadings in mouse tissues

At 12 h post infection, the diseased mice were selected for euthanization and dissection. The right hemisphere of the brain, lungs and blood samples were aseptically collected and homogenized with sterile pre-cooled PBS. Bacteria were isolated from the abovementioned homogenates by plating 10-fold serial dilutions on MacConkey plates. The bacterial loadings were calculated by CFU per gram of organs or per microliter of blood.

### Evans blue permeability assay

Evans blue (EB) can penetrate the blood-brain barrier (BBB) when the integrity of BBB is disrupted under a pathological state. So, the integrity and permeability of BBB can be evaluated by measuring the EB amount in the brain. A 2% EB solution in normal saline (100 μL per mouse) was injected into the caudal vein of mice. After 30 min, the mice were anesthetized and perfused with 50 mL of ice-cold PBS transcardially. After euthanasia, brain tissues were collected and homogenized in 1.1 mL of pre-cooled PBS and then centrifuged at 15,000 g for 30 min at 4°C [21]. The supernatant was collected in aliquots. Each 500 μL of supernatant was mixed into an equal amount of 50% trichloroacetic acid (Enox, China), incubated for 12 h at 4°C, and centrifuged at 15,000 g for 30 min at 4°C to separate the supernatants. The absorbance was measured at 630 nm using a spectrophotometer.

### Expression of ZO-1, occludin, and claudin-5 proteins examination by western blot

Expressions of zonula occludens (ZO)-1, occludin, and claudin-5 proteins in brains were measured by western blot. Briefly, total proteins were extracted from the brains using RIPA Lysate Buffer (Beyotime Biotechnology) and separated by 12% SDS–PAGE. Gels were blotted onto PVDF membranes using a Trans-Blot SD system (Bio-Rad, Hercules, CA, USA). The membranes were rinsed with TBST20 buffer (20 mM Tris base, 150 mM NaCl and 0.1% Tween 20), blocked for 2 h in 10% skimmed milk and incubated with primary antibodies overnight at 4°C, including ZO-1 (1:1000; Invitrogen, Carlsbad, CA, USA), occludin (1:500; Invitrogen), claudin-5 (1:50; Invitrogen) and GAPDH (1:1000; Cell Signaling Technology). The blots were washed three times with TBST20 buffer. Then, the membranes were incubated with HRP-conjugated secondary antibodies (1:10000 dilution in 5% skimmed milk) at room temperature for 1 h. The blots were washed three times with TBST20 and developed using an enhanced chemiluminescence for 30 s. The blot intensity was analyzed using a chemiluminescence imaging system (Clinx Science Instruments, ChemiScope 5300, China).

### Detection of inflammatory cytokine expression in brain tissues by quantitative real-time PCR (qRT-PCR)

Total RNA from brain tissues was extracted using TRIzol reagent (Invitrogen). cDNA was synthesized using the PrimeScript RRT reagent kit with gDNA Eraser according to manufacturer protocol (Takara, Tokyo, Japan). Primers for amplifying *IL-1β*, *IL-6* and tumor necrosis factor-α (*TNF-α*) are listed in S1 Table. Relative transcript abundance was determined by qRT-PCR with SYBR Premix Ex Taq II (Takara, Tokyo, Japan) using an ABI7500 instrument (Applied Biosystems, Foster, CA, USA). Assays were performed in triplicate, and all data were normalized to the endogenous reference gene *GAPDH* using the 2^-△△CT^ method.

### Magnetic resonance imaging (MRI) scanning

The MRI scanning of brains was performed on a 7.0-T MRI scanner (Bruker Corporation, BRUKER BIOSPEC 70/30, Germany). 5 mice in each group were used for Magnetic resonance imaging (MRI) scanning. After 12 h post infection, the mice were anaesthetized by 2% isoflurane inhalation and then maintained with 1.5% isoflurane. Then, the head of mouse was fixed with two flat head plastic thumbscrews and placed on a heating pad for maintaining the body temperature at 37°C. The saturation of pulse oxygen, heart rate, respiratory rate and rectal temperature of mouse were monitored during the scanning. The brain was scanned using mapping sequence and T1-weighted imaging (T1WI) sequence. Gadopentetate meglumine contrast was given by tail vein injection at the dosage of 0.1 mmol/kg of a 100 μL solution. Then the contrast-enhanced T1WI sequence was performed to determine the permeability changes of BBB after infection.

### Brain histopathology and immunohistochemical analysis

The brain samples were excised and fixed in 4% paraformaldehyde for 2 d. Then, the tissues were dehydrated using serial gradient alcohol and xylene and embedded in paraffin. The embedded tissues were cut into 4-μm paraffin sections by an automated microtome (Leica, Weztlar, Germany) and stained with hematoxylin and eosin (H&E). The brain sections were observed and analyzed using a microscope (Nikon, Eclipse 80i, Japan). Additionally, serial sections were used for immunohistochemical analysis. Briefly, the active endogenous peroxidase was blocked by 3% hydrogen peroxide. The sections were placed in citrate buffer at 100°C for 15 min, incubated with 5% bovine serum albumin (BSA; Boster Biological Technology) at 37°C for 1 h and subsequently incubated with primary antibodies against ZO-1 (1:100 dilution; Invitrogen), occludin (1:100; Invitrogen) and claudin-5 (1:200; Invitrogen) overnight at 4°C. The sections were washed and incubated with secondary antibody for 45 min at room temperature. After washing with PBS three times, the sections were stained with 0.1% 3, 3’-diaminobenzidine (DAB; Boster Biological Technology) and counter-stained with hematoxylin. Protein expression was observed with a microscope (Leica), and images were analyzed by ImageJ software.

### Statistical analysis

Data were analyzed with SPSS 17.0 software (SPSS, Chicago, IL, USA) using one-way ANOVA for multiple comparisons. Differences were considered significant when *p* ≤ 0.05. The results of immunohistochemical analysis were analyzed with Image-Pro Plus 6.0. Three biological replicates were used in each experiment with three technical replicates.

## Results

### *ClbA* deletion does not affect the growth of APEC XM

The effect of ClbA on the growth of APEC was analyzed by the growth curves. The growth of the *clbA* mutant in LB liquid medium was similar to that in WT and complemented mutant (S2 Fig). This indicated that the deletion of *clbA* did not affect the growth of APEC XM.

### ClbA is involved in colibactin production and elicits genotoxic effects in vitro

The γH2AX expression and megalocytosis level in bEnd.3 cells were detected to evaluate the effect of ClbA in colibactin production and genotoxicity. The result of immunofluorescence staining assay of γH2AX showed that, after 4 h of infection, the percentages of γH2AX positive cells infected by APEC XM Δ*clbA* decreased significantly at 0 h post-infection (hpi, S3A and S3B Fig) and 72 hpi (S3C and S3D Fig) (*p*<0.01) when compared with that infected by APEC XM. There were no differences in the phosphorylation of H2AX between the APEC Δ*clbA* infection group and the control group. This indicated that deletion of *clbA* led to a decreased ability of APEC to damage DNA in bEnd.3 cells. The APEC XM Δ*clbA*/p*clbA* completely restored the genotoxicity to bEnd.3 cells.

Colibactin led to megalocytosis in bEnd.3 cells due to blocking of the cell cycle. Megalocytosis was measured by methylene blue staining and absorbance value determination at 630 nm. The cells infected with AEPC XM Δ*clbA* showed attenuated megalocytosis and significantly higher absorbance (630 nm) compared with the AEPC XM infection group (*p*<0.01) but a similar absorbance as the control group (S3E Fig). This indicated that ClbA affected the genotoxicity to bEnd.3 cells and colibactin production of AEPC XM. The APEC XM Δ*clbA*/p*clbA* completely restored colibactin production ability.

### ClbA plays an important role in the virulence of APEC XM in vivo

**Fig 1:**
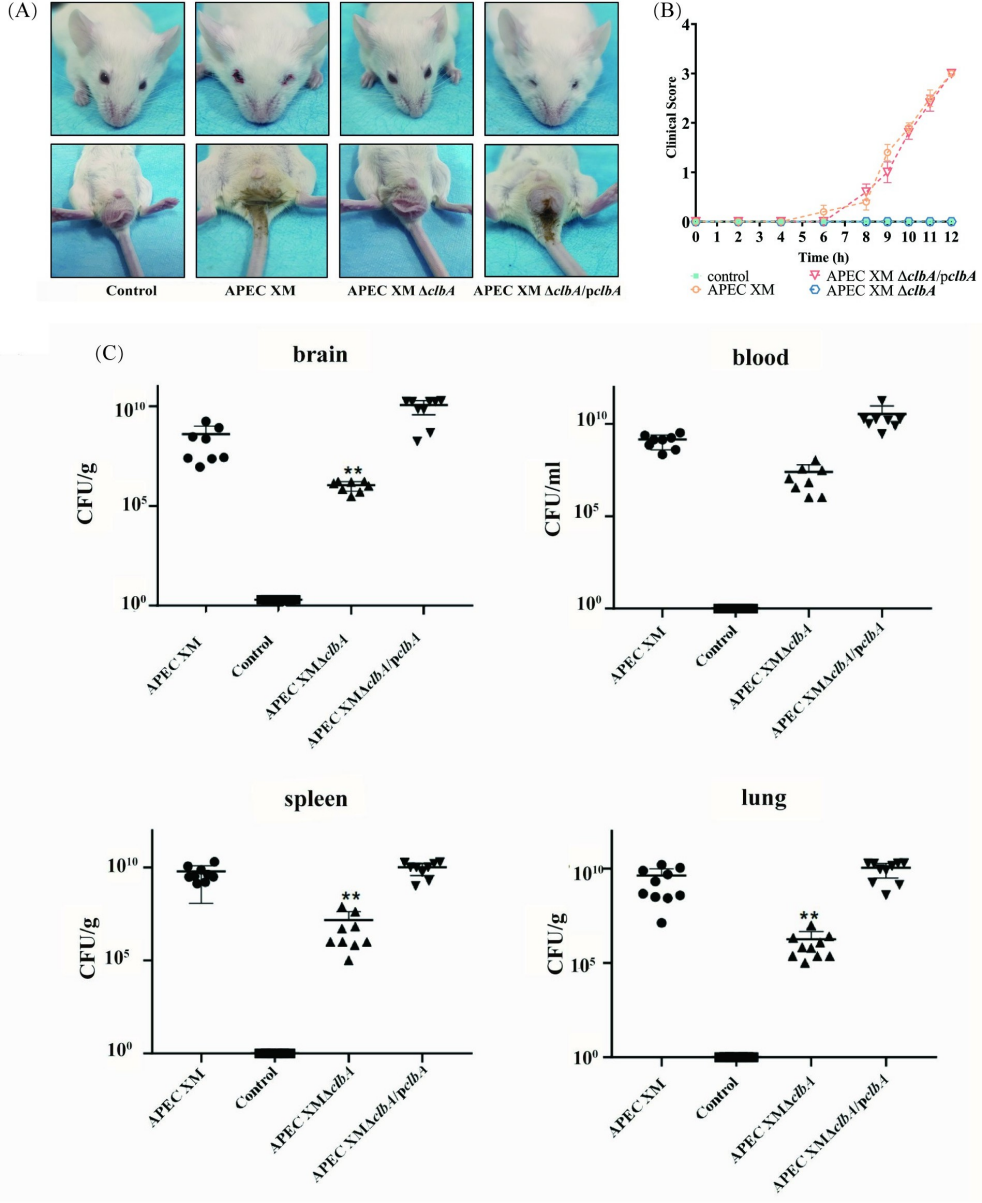
ClbA is required for the virulence of APEC XM *in vivo*. (A) Clinical symptoms of mice after 12h infection. The mice infected by APEC XM suffered from lethargy, thick eye discharge and diarrhea. The mice infected by APEC XMΔ*clbA* displayed very mild symptoms. (B) Clinical scores of mice which challenged with APEC XM, APEC XMΔ*clbA* and APEC XMΔ*clbA*/p*clbA* for 12h infection. Ten mice in each group were used this experiment. The clinical scores were recorded at time points of 2, 4, 6, 8, 9, 10, 11, 12 hour. Data were analyzed with one-way ANOVA and presented as the mean±standard errors of the mean for the clinical scores of 10 mice in each group. (C) Bacterial loads in the brain, blood, spleen and lungs of infected mice after 12 h of infection. 10 mice in each group were used in this experiment. Dots represent the values of bacterial loads in the organs of each infected mouse which are calculated by plate counting with triplicate replicates. Data were analyzed with one-way ANOVA and presented as the mean±standard errors of the mean for the organs of 10 mice. Significant differences between the APEC XMΔ*clbA* and wild-type strain APEC XM are indicated (**p < 0.01, versus APEC XM group).

The ClbA function in the virulence of APEC XM was evaluated by infecting a mouse model with wild-type APEC XM, APEC XMΔ*clbA* and APEC XMΔ*clbA*/p*clbA*, respectively. After challenge with APEC XM for 6 h, the mice began to show clinical signs one after another. Especially during 10–12 hours, most mice displayed serious clinical symptoms such as depression, thick eye discharge, diarrhea, neurological symptoms and unable to walk (clinical score 2~3, Fig 1A and 1B). At 12h, clinical scores of all the mice reached 3. In the APEC XMΔ*clbA* infection group, the mice did not present apparent behavioral abnormality (clinical score 0, Fig 1A and 1B), while the mice infected by APEC XMΔ*clbA*/p*clbA* displayed similar clinical signs with APEC XM infected mice (Fig 1A and 1B).

The bacterial loads in the brain, spleen and lungs were significantly decreased in the APEC XMΔ*clbA* infection group (*p*<0.01), when compared with the APEC XM infection group. In the APEC XMΔ*clbA*/p*clbA* infection group, the bacterial loads in the above mentioned organs were similar to those in the APEC XM infection group (Fig 1C). These results indicated that the deletion of *clbA* decreased the virulence of APEC XM, and the complementation of *clbA* restored the virulence of APEC XM.

### ClbA contributes to blood-brain barrier disruption in vivo

**Fig 2:**
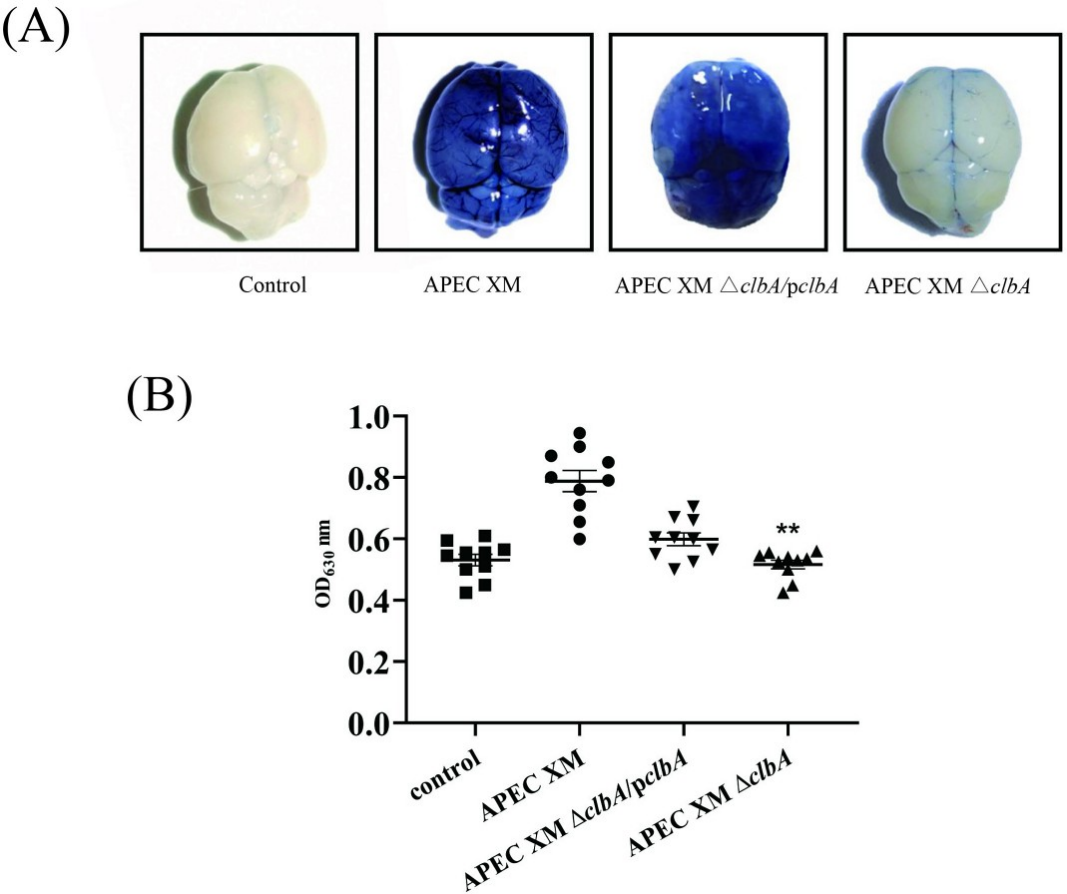
Determination of BBB integrity by EB staining. 10 mice in each group were inoculated intraperitoneally with 10^7^ CFU of test bacteria (APEC XM, APEC XMΔ*clbA*, and APEC XMΔ*clbA*/p*clbA*) or with an equal volume of sterile saline. After 12 h of infection, 2% EB solution was injected to the mice. EB content that penetrated the brain was determined by measuring the absorbance (OD_630_) after brain homogenization and precipitation. (A) Dorsal view of Evans blue stained mouse brains after EB injection; (B) Quantification of EB content in mice brain. Dots represent the OD_630_ values of EB content in the brains of each infected mouse. Data were analyzed with SPSS 17.0 software using one-way ANOVA. Error bars represent the standard errors of the means. Significant differences between the wild-type strain APEC XM and the APEC XMΔ*clbA* are indicated (**p < 0.01, versus APEC XM group).

The amount of EB in the brain is proportional to the integrity of BBB. In this study, the brains of mice infected by APEC XM, APEC XMΔ*clbA*, and APEC XMΔ*clbA*/p*clbA* were stained by Evans blue dye. Compared to the negative control group, significant EB stain accumulation was observed in the mice brains infected by APEC XM and APEC XMΔ*clbA*/p*clbA*, while only slight stain accumulation was observed in the brains of APEC XMΔ*clbA*-infected mice (Fig 2A). Quantification of EB in the APEC XMΔ*clbA* infection group was significantly decreased, compared with the APEC XM infection group (*p*<0.01) (Fig 2B). There was no significant difference in the quantification of EB between the APEC XM and APEC XMΔ*clbA*/p*clbA* infection groups (Fig 2B).

**Fig 3:**
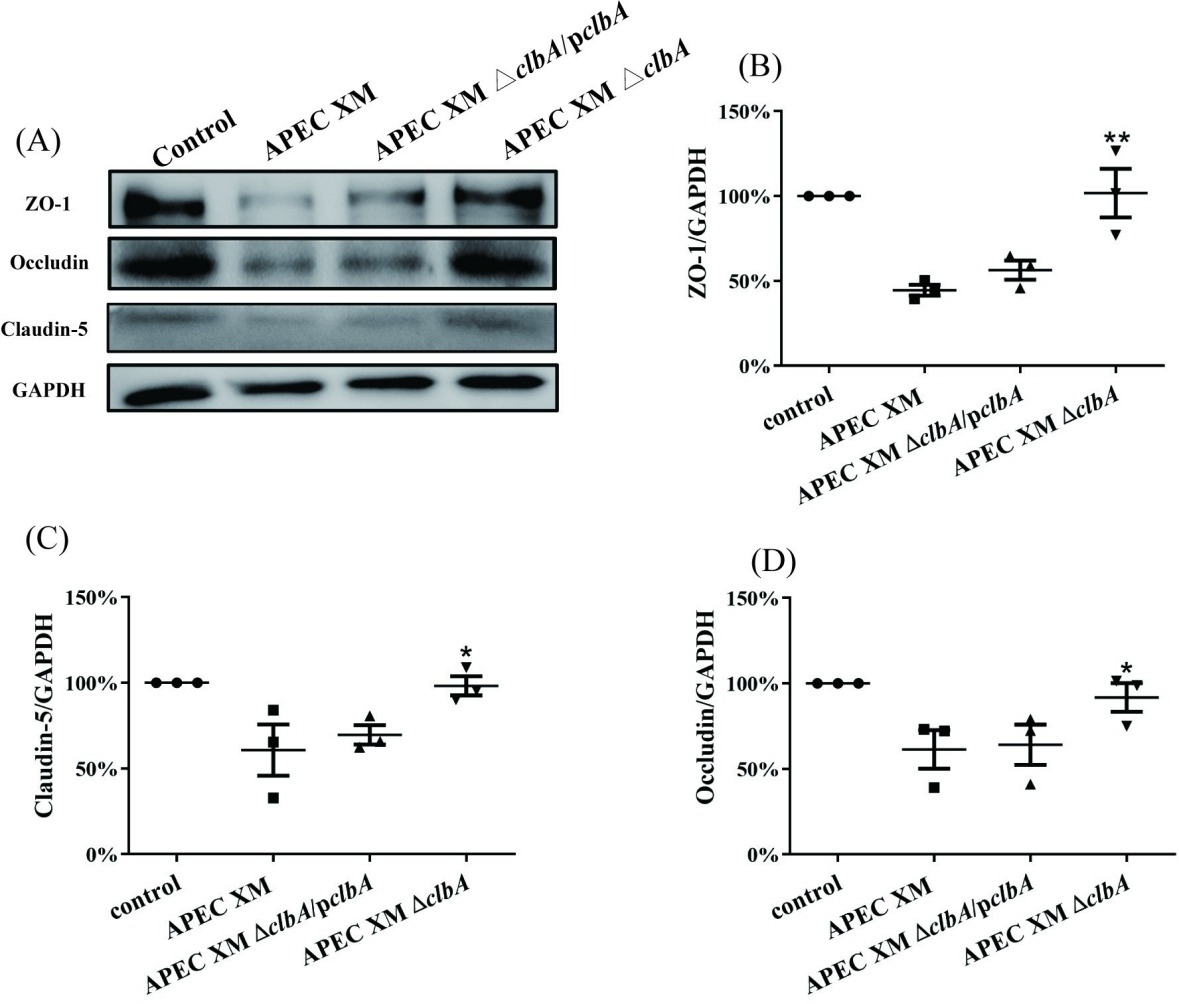
The expressions of ZO-1, claudin-5 and occludin in the brain were measured by western blot. 9 mice in each group were used in this experiment. Because of low protein content, samples from each of the three mice in each group were merged together as one sample for WB detection. In other words, this experiment was repeated three times with three technical replicates. GAPDH was used as a loading control. The protein expression in the control group is defined as 100%. The dots represent the ratio of gray density value of protein in bacterial infection group to that in control group for each replicate. Data are analyzed with SPSS 17.0 software using one-way ANOVA and expressed as mean ± standard error of the mean for triplicate experiments. Significant differences between the wild-type strain APEC XM and the APEC XMΔ*clbA* are indicated (**p < 0.01, *, 0.01 < *p* < 0.05, versus APEC XM group).

**Fig 4:**
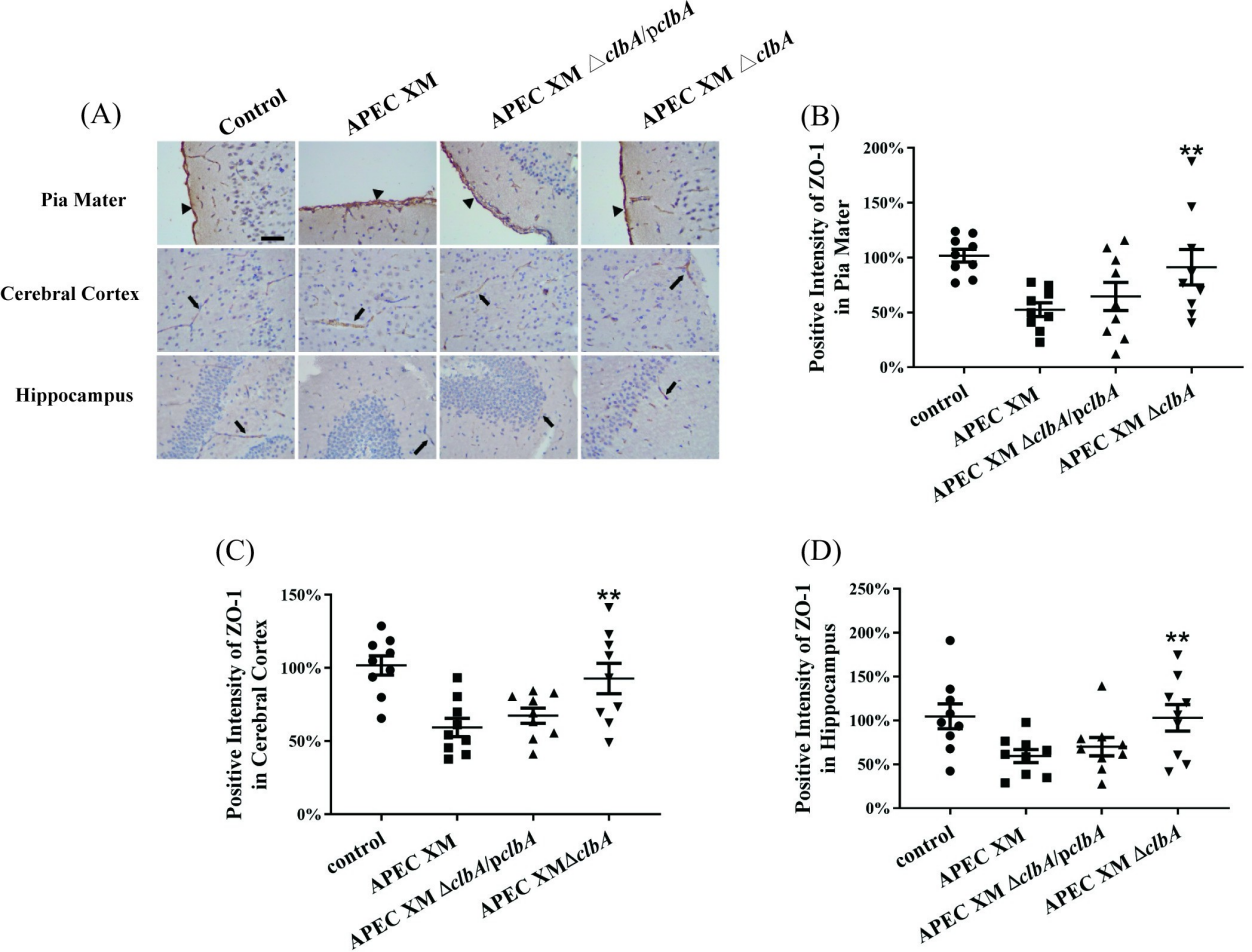
The expressions of ZO-1 in the brain were measured by immunohistochemistry. This experiment was repeated three times. 3 mice were used in each group for each experiment. (A) Immunohistochemistry staining of ZO-1 protein in infected mouse brains (bar = 20 μm). The black arrowheads point to the pia maters, and the black arrows point to the microvascular. The percentage of positive staining intensity of ZO-1 in pia mater (B), cerebral cortex (C) and hippocampus (D) was calculated by defining the relative optical density of the control group as 100%, and the relative optical densities of other groups were normalized by the control group. The dots represent the relative optical densities for each replicate. Data are analyzed with SPSS 17.0 software using one-way ANOVA. The results are presented as the mean ± standard deviations of three independent experiments. Significant differences between the wild-type strain APEC XM and the APEC XMΔ*clbA* are indicated (**, *p* < 0.01, versus APEC XM group).

**Fig 5:**
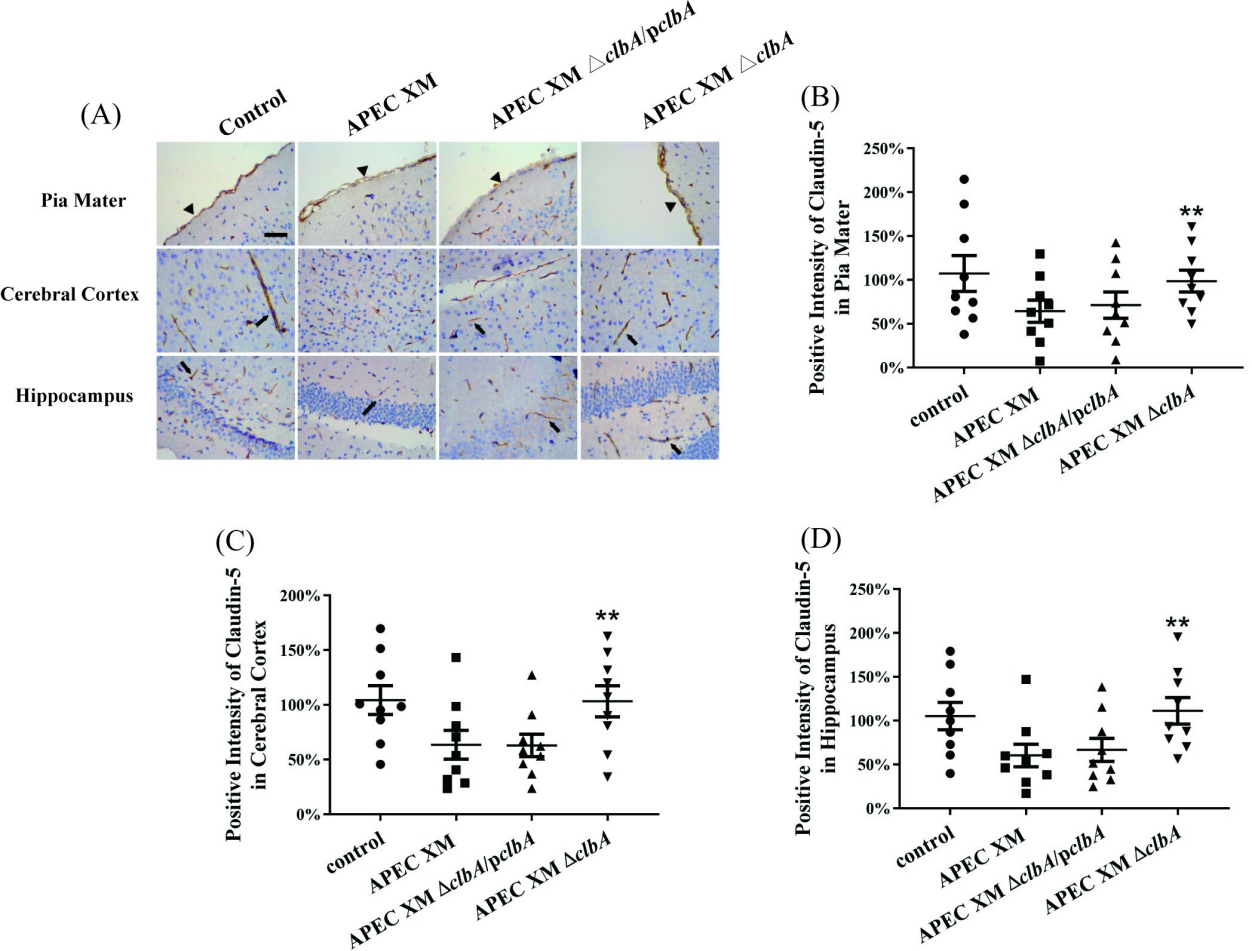
The expressions of claudin-5 in the brain were measured by immunohistochemistry. (A) Immunohistochemistry staining of claudin-5 protein in infected mouse brains (bar = 20 μm). (B-D) The percentage of positive staining intensity of claudin-5 in pia mater (B), cerebral cortex (C) and hippocampus (D). The methods of positive staining intensity calculation and data analysis were same as that in Fig 4.

**Fig 6:**
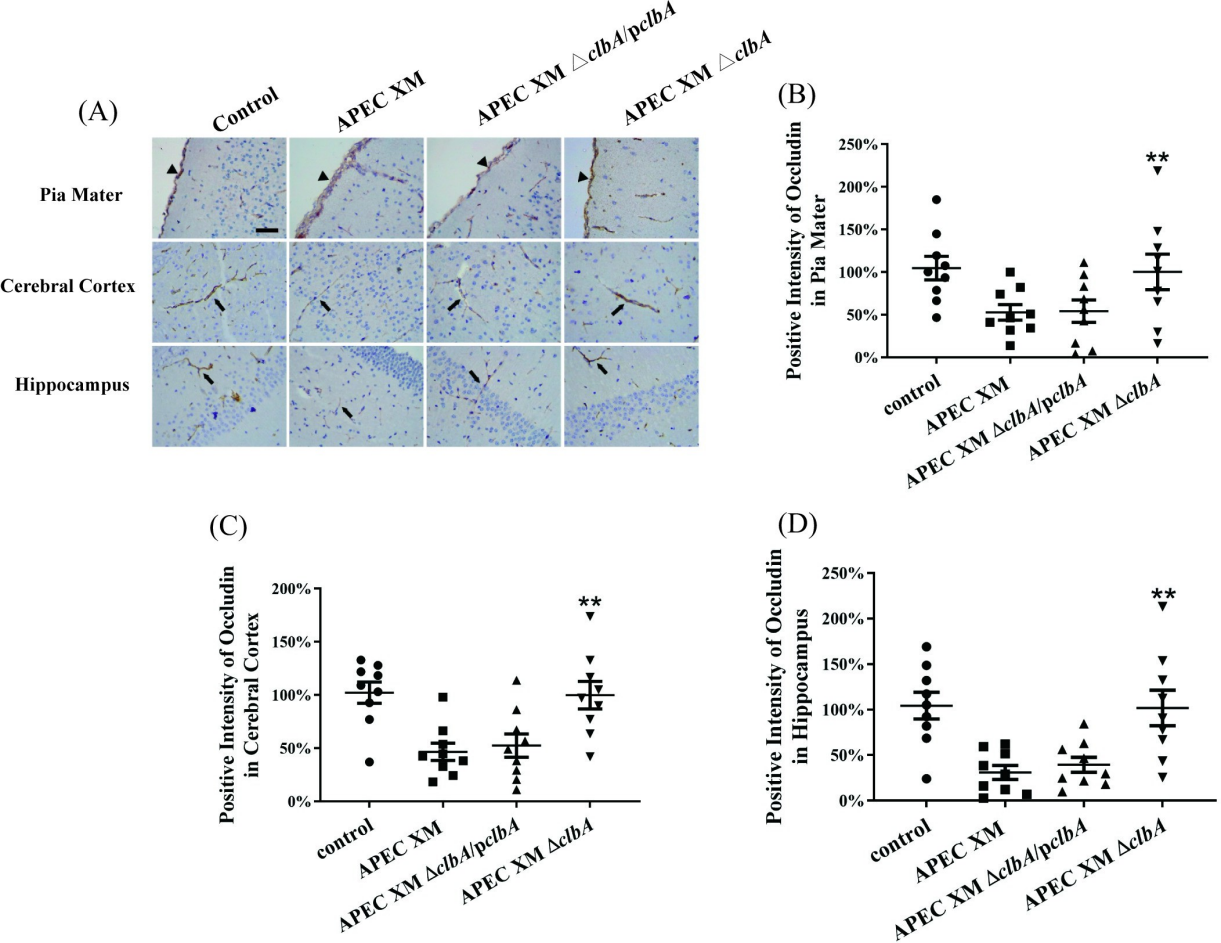
The expressions of occludin in the brain were measured by immunohistochemistry. (A) Immunohistochemistry staining of occludin protein in infected mouse brains (bar = 20 μm). (B-D) The percentage of positive staining intensity of occludin in pia mater (B), cerebral cortex (C) and hippocampus (D). The methods of positive staining intensity calculation and data analysis were same as that in Fig 4.

The expressions of tight junctional proteins ZO-1, occludin and claudin-5 were detected *in vivo* by western blot and immunohistochemical staining. The western blot results showed that, compared with the negative control group, the expressions of ZO-1, occludin and claudin-5 in the brains of mice infected by APEC XM and APEC XMΔ*clbA*/p*clbA* were all significantly decreased, while the expression level in the APEC XMΔ*clbA* infection group was similar to that in the control group (Fig 3A–3D). The immunohistochemical staining of ZO-1 (Fig 4A), claudin-5 (Fig 5A) and occludin (Fig 6A) can be observed in the pia mater, cerebral cortex and hippocampus of the mice brains. Compared with the negative control group, the expression of ZO-1 (Fig 4B–4D), claudin-5 (Fig 5B–5D) and occludin (Fig 6B–6D) in the pia mater, cerebral cortex and hippocampus of mice brains infected by APEC XM and APEC XMΔ*clbA*/p*clbA* was significantly decreased, while the expression level in the APEC XMΔ*clbA* infection group was similar to that in the control group. These results indicated that deletion of *clbA* decreased the disruption of BBB integrity and permeability.

### ClbA contributes to brain lesions in mice

**Fig 7:**
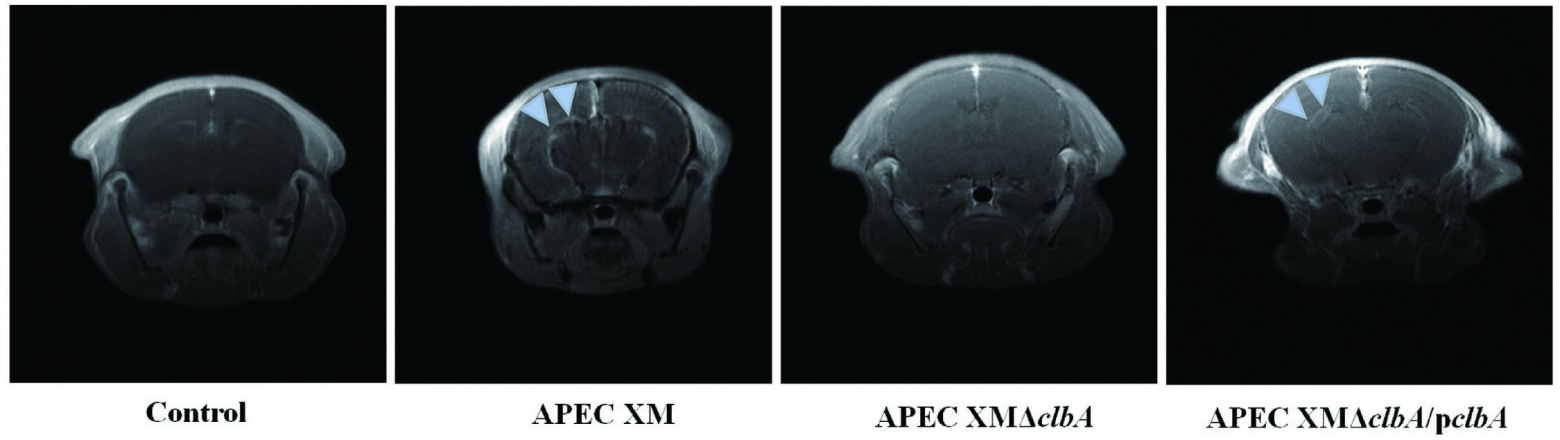
Lesion examination of mouse brains by MRI scanning. MRI was performed to assess the brain lesions at 12 h post infection. Diffusion enhancement of the cerebral parenchyma (Blue Arrowhead) are observed in the mice infected with APEC XM or APEC XMΔ*clbA*/p*clbA*.

The MRI scan (enhanced T1WI) revealed that a range of lesions such as thickened pia mater, widened sulci and a diffusion enhancement of cerebral parenchyma occurred in mice brains infected by APEC XM. The lesion characteristics of mice infected by APEC XMΔ*clbA* were lower when compared with the APEC XM infection group. The lesion causing ability of APEC XMΔ*clbA*/p*clbA* restored partly (Fig 7). It indicated that ClbA contributed to brain lesions in mice.

**Fig 8:**
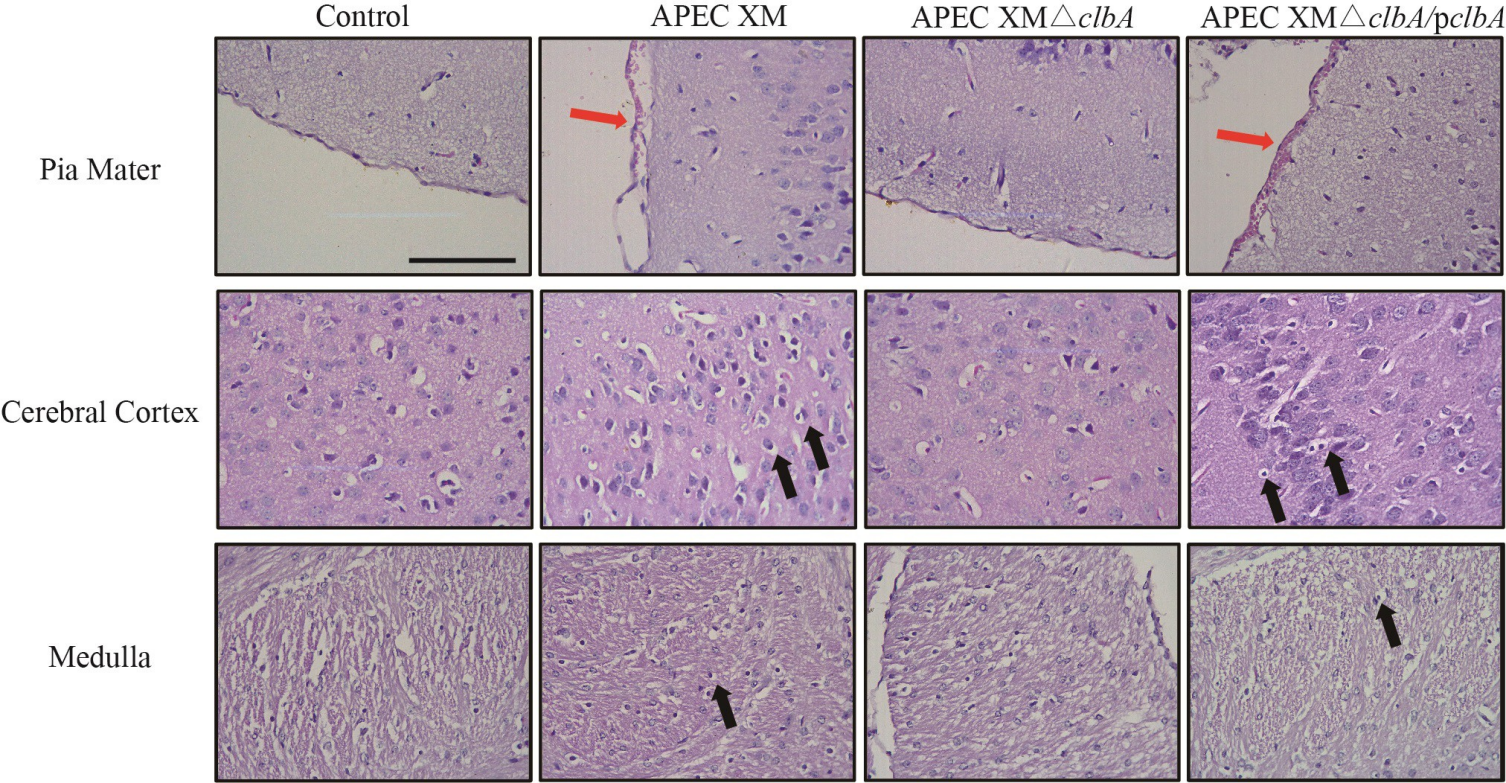
Histopathological analysis of the brain tissue of 4-week-old ICR mice infected with APEC XM, APEC XMΔ*clbA* and APEC XMΔ*clbA*/p*clbA* and non-infected mice. Sections were stained with hematoxylin-eosin and visualized with an optical microscope (bar = 100 μm). Thickened pia mater with hemorrhage (red arrowheads) was observed in the brain section of mice infected by APEC XM and APEC XMΔ*clbA*/p*clbA*. Leukocyte infiltration (black arrowheads) was observed in the cerebral cortex and medulla of mice infected with APEC XM and APEC XMΔ*clbA*/p*clbA*.

The histopathological analysis showed that pia mater of the brains in mice challenged with APEC XM was discontinuous, edematous and was detached from the cerebral cortex. Leukocyte infiltration could be observed in the cerebral cortex. There were no obvious histopathological characteristics in the brains of mice when challenged with APEC XMΔ*clbA*. The mice brains challenged with APEC XMΔ*clbA*/p*clbA* showed histopathological characteristics similar to those in the mice infected by the wild-type strain (Fig 8).

**Fig 9:**
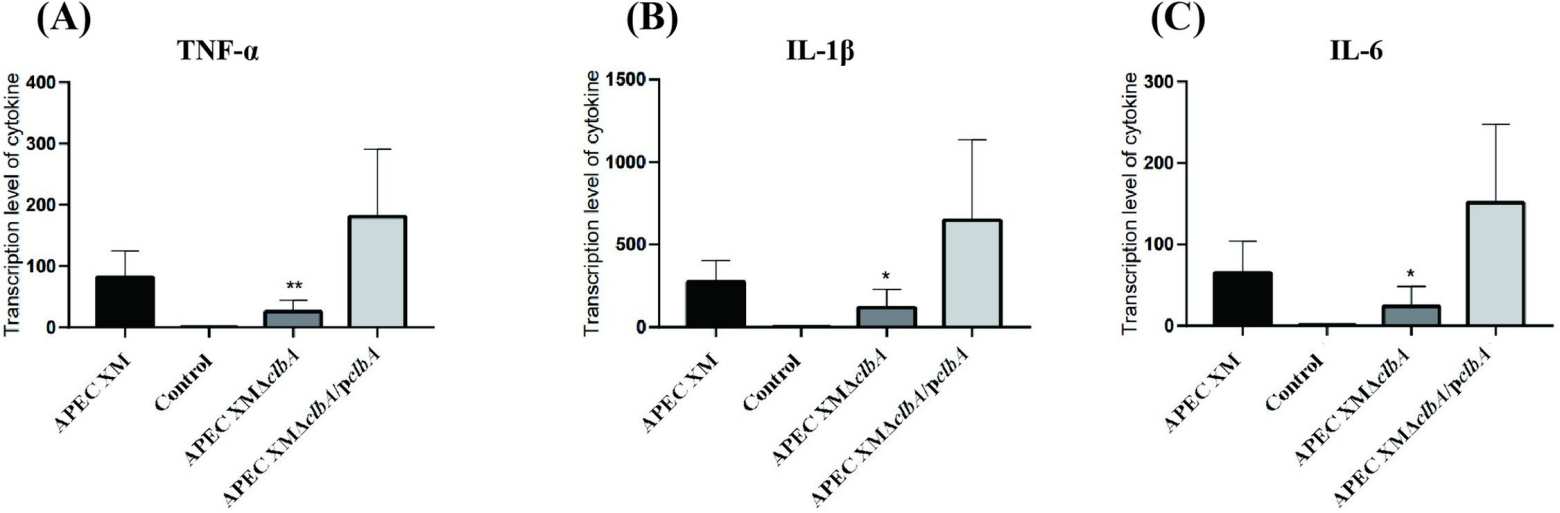
The mRNA transcript levels of inflammatory cytokines *TNF-α* (A), *IL-1β*(B), and *IL-6* (C) were determined by qRT-PCR. This experiment was repeated three times. 3 mice were used in each group for each experiment. The relative gene expression level was calculated using the 2^-△△^CT method. *GAPDH* was used as the normalizing internal standard. The data were analyzed with SPSS 17.0 software using one‐way ANOVA and presented as mean ± standard deviations of three replicates. Significant differences between APEC XMΔ*clbA* and APEC XM are indicated (**, *p* < 0.01, *, 0.01 < *p* < 0.05, versus APEC XM group).

The relative expressions of *IL-1β*, *IL-6* and *TNF-α* mRNA in brain tissues were measured by qRT-PCR. Compared with the negative control group, the relative expressions of *IL-1β*, *IL-6* and *TNF-α* were all increased significantly in the brains of mice infected by APEC XM (*p*<0.01). *IL-1β*, *IL-6* and *TNF-α* mRNA expression was decreased in the APEC XMΔ*clbA* infection group compared with the APEC XM infection group (*p*<0.01), while there was no significant difference between the APEC XMΔ*clbA*/p*clbA* and APEC XM infection groups (Fig 9). Based on the results of the MRI scan, histopathological analysis and inflammatory cytokine expression, we conclude that the deletion of *clbA* decreased the brain lesions caused by APEC XM in mice.

## Discussion

APEC XM (O2:K1) used in this study induces meningitis in animals and is considered to have potential zoonotic risk. Because APEC and NMEC utilize similar pathogenic strategies for causing meningitis [22], the meningitis-causing mechanism of APEC is attracting increasing attention. The development of meningitis is dependent on a series of virulence factors such as IbeA and OmpA. However, many factors are uncharacterized. Colibactin, encoded by a *pks* island, is produced in many B2 phylogenetic group *E*. *coli* strains that cause sepsis and meningitis in human neonates [23–25]. Phylogenetic analysis revealed that the majority of meningitis *E*. *coli* belonged to the B2 phylogroup [26]. ClbA, a PPTase, contributes to the production of both colibactin and PPTase-dependent siderophores [6, 12]. It is unclear whether ClbA has a pleiotropic function in virulence and meningitis-causing ability of APEC. In this study, we established a mouse infection model, abolished the *clbA* gene and evaluated the pathogenic role of ClbA in meningitis caused by APEC XM.

In our study, the established mouse infection model is suitable for evaluating the function of ClbA on meningitis development. Although APEC XM was originally isolated from the brains of ducks, this strain could also infect mice [14]. APEC is considered to be a potential pathogen of humans because of the high similarity between APEC and NMEC [22]. A mouse model is valuable to clarify the potential pathogenic mechanism in mammals. We found that APEC XM could infect mice, and the infected mice displayed a series of typical clinical symptoms associated with meningitis. This indicated that the model was constructed successfully and could be used for further study of the ClbA function in meningitis development.

In *E*. *coli* K1 and *Klebsiella pneumoniae*, ClbA contributes to the synthesis of both colibactin and siderophores [12, 13]. Colibactin is a genotoxin that induces DNA damage and chromosomal abnormalities *in vitro* and *in vivo* [7, 27]. Colibactin is a veritable virulence factor in the mouse model with sepsis or neonatal meningitis. *Pks*-harboring *E*. *coli* could be isolated from immunocompromised mice with urosepsis and meningitis [28]. The production of colibactin by septicemic *E*. *coli* exacerbates lymphopenia and decreases the survival rate in mice [29]. In an *E*. *coli* strain expressing the K1 capsule, colibactin contributes to the colonization capacity of the neonatal gastrointestinal tract and subsequently causes systemic infection in neonates [9]. Abolishment of colibactin production hinders *K*. *pneumoniae* hypervirulence in key pathogenic steps toward meningitis development [13]. It indicated that colibactin was an important virulent factor in *K*. *pneumoniae* and *E*. *coli*. Siderophore-mediated iron acquisition is important for the survival of *E*. *coli* in the host and extra intestinal infection. As a critical factor for siderophore synthesis, ClbA contributes to the virulence of *E*. *coli* during the step of the infection in a mouse model of sepsis [12]. Moreover, *clbA* was essential for colibactin production and substantially attenuated the genotoxicity to mammalian cells [6, 9, 13].Our study revealed that the *clbA* mutation led to the loss of genotoxicity of APEC XM to bEnd.3 cells. This indicated that ClbA was a critical factor for colibactin synthesis. Besides *clbA*, all the genes in *pks* island were required to synthesize colibactin in *E*. *coli* [6]. Previous studies showed that the deletion of *clbG* or *clbH* abolished the production of colibactin and then affected the development of meningitis in mice induced by APEC [30, 31]. This study demonstrated that the deficiency of *clbA* significantly attenuated APEC virulence in systemic infections and the process of meningitis development including bacteremia, penetration of the BBB and induction of pro-inflammatory factor expression. The decrease of virulence caused by *clbH* or *clbG* deletion was much less obvious than that caused by *clb*A deletion. It was speculated that the contribution of ClbA in attenuation of APEC virulence was caused by not only colibactin-caused cytotoxicity but also ClbA-mediated iron uptaken.

Bacterial meningitis usually develops through several processes. These include mucosa colonization in the gastrointestinal tract or upper respiratory tract, crossing the mucous membrane cell layer, invasion into and survival/multiplication in the bloodstream with a high level of bacteremia, traversing the BBB and entry into the subarachnoid space. After these processes, bacterial meningitis elicits inflammation and pathophysiological alterations such as BBB disruption and brain damage [32–34]. Multiplication in the bloodstream to reach the threshold level of bacteremia is necessary for meningitis development [32]. A significantly higher occurrence of *E*. *coli* meningitis in neonates is observed when the bacterial counts in blood are higher than 10^3^ CFU/mL [35]. In this study, mice infected by APEC XM had more than 10^5^ CFU/mL bacterial loads in the blood, and they suffered from meningitis. In addition to their presence in the blood, bacteria were also isolated from the brain, spleen and lungs of mice. This indicated that APEC XM caused a systemic infection. Deletion of *clbA* significantly reduced the bacterial loads of APEC XM in the blood, brain, spleen and lungs of mice. It indicated that ClbA and colibactin in APEC were important in the bacteremia and systemic infection of mice. Colibactin in the *E*. *coli* K1 strain also contributed to invasion of the blood and was associated with bacteremia and systemic infection in a neonatal rat model [9]. We speculate that colibactin in neonatal meningitis-causing *E*. *coli* and that in APEC have similar pathogenic mechanisms in the bacteremia and systemic infection.

Penetration of the BBB is a prerequisite for the development of central nervous system (CNS) infection and meningitis [36, 37]. EB staining of brain showed that the permeability of BBB in the mouse brain was enhanced by APEC infection. Deletion of *clbA* significantly reduced the ability of APEC to penetrate the BBB. ZO-1, occludin and claudin-5 are the main elements of intercellular tight junction proteins, which are responsible for the BBB structure and functional integrity [38]. Damage to, or detachment of ZO-1 may enhance the barrier permeability. Occludin is a tight junction-associated transmembrane protein with barrier function [39]. Claudin-5 is an important component of the tight junctions by selectively decreasing the permeability to ions and maintaining high transepithelial electrical resistance [40]. Previous studies demonstrated that the expressions of ZO-1, occludin and claudin-5 were reduced in the brain endothelial cells infected by *E*. *coli* [41] or *Neisseria meningitidis* [42]. We demonstrated that the expressions of ZO-1, occludin and claudin-5 were observably decreased when the mice were infected by APEC XMΔ*clbA*, compared with mice infected by APEC XM. It indicated that *clbA* deletion in APEC reduced BBB disruption in the brain of mice. Due to a pleiotropic phenotype of *clbA* mutant related to colibactin and siderophores, this reduction of BBB disruption could be caused by a direct or indirect combined effects of colibactin and siderophores. The previous study showed that colibactin contributed to serum resistance ability of APEC [30]. Deletion of colibactin-encoded genes such as *clbH*, *clbG* decreased the bacterial load in the blood of APEC infected mice [30, 31]. *ClbA* deletion also showed the similar phenotype in our study. It was supposed that colibactin-mediated bacterial serum resistance and the amount of bacteria in the blood led to the reduction of BBB disruption. Moreover, as a genotoxin, colibactin probably reduced the BBB disruption by inducing DNA damage in mouse brain microvascular endothelial cells. In addition, ClbA could contribute to siderophores synthesis. The presence of ClbA was required for the survival of ExPEC in vivo [12]. Reduction of BBB disruption in *clbA* mutant could be caused by a non-specific damage of the tissue associated with increased survival of the bacteria in the extraintestinal compartment.

Once bacteria enter the CNS, they multiply and induce brain dysfunction and the release of host pro-inflammatory factors such as cytokines IL-1β, IL-6 and TNF-α [43]. IL-1β is associated with systemic inflammatory response and contributes to macrophage recruitment and *Streptococcus pneumoniae* clearance [44]. TNF-α is a powerful inflammatory cytokine related to the acute phase of inflammation. TNF-α and IL-6 can induce a permeability increase of BBB, which is caused by a decrease of ZO-1 and occludin expression and loss of BBB integrity [45, 46]. The infection caused by APEC in this study led to a significant increase in the expression of IL-1β, IL-6 and TNF-α mRNA in mice brains. The increased expression of these cytokines is consistent with the characteristics of bacterial meningitis. The cytokine expressions were decreased because of *clbA* deletion in APEC. Histopathological analysis and MRI examination also showed that deletion of *clbA* reduced the brain injuries of mice infected by APEC. This indicated that colibactin contributes to the pathogenicity of APEC in brain damage and meningitis development.

In summary, ClbA is an essential factor for colibactin production and contributes to APEC virulence in systemic infections and meningitis development in mice. However, the manner in which ClbA affect meningitis development through genotoxicity and iron absorption remains unclear. Because colibactin has not been purified [7], the direct toxic effect of colibactin on the mouse brain remains unknown.

## Supporting information

S1 Table Primers used for mutant construction and qRT-PCR. (PDF)

S1 Fig PCR verification of the mutant APEC XMΔ*clbA* and its complementation APEC XMΔ*clbA*/p*clbA*. The mutants were identified by PCR amplification using primers ClbA -VF and ClbA-VR. About a 1230bp PCR product was obtained when the wild-type APEC XM genome as template (lane 1). APEC XMΔ*clbA* mutant was verified by amplifying a 260bp fragment (lane 2). Both a 1230bp fragment and a 260bp fragment were obtained in APEC XMΔ*clbA*/p*clbA* (lane 3). M: DL2000 DNA Marker.
(TIF)

S2 Fig Growth curves of APEC XM, APEC XMΔ*clbA*, and APEC XMΔ*clbA*/p*clbA*. OD_600 nm_ values of triplicate cultures in LB medium were determined at 1h intervals. Data are the means of three independent experiments.
(TIF)

S3 Fig ClbA is required for colibactin synthesis and genotoxic effects of APEC XM on bEnd.3 cells. Immunofluorescence staining assay of γH2AX protein in bEnd.3 cells at 0 hpi (A) and 72 hpi (C). The nuclear DNA and γH2AX are colored blue and green, respectively (bar = 200 μm). Quantification of DNA double-strand breaks (DSBs) by calculating the percentages of γH2AX positive cells within the total cells at 0 hpi (B) and 72 hpi (D), normalized to the APEC XM infection group. The ratio of the APEC XM group was set as 100%. (E) Megalocytosis was quantified by determining the absorbance values at 630 nm. The results are given as mean ± standard error and analyzed with one‐way ANOVA (**, *p* < 0.01, versus APEC XM group). All assays were performed with three independent biological replicates.
(TIF)

S1 Raw images (PDF)

S2 Raw images (PDF)
