# Supplementary material for: Phosphopantetheinyl transferase ClbA contributes to the virulence of avian pathogenic Escherichia coli in meningitis infection of mice
Source: PLoS One. 2022 Jul 28;17(7):e0269102. doi: 10.1371/journal.pone.0269102 (PMC9333332; doi:10.1371/journal.pone.0269102)
Supplement: S1 Table — (PDF) [file pone.0269102.s001.pdf]

S1 Table Primers used for mutants construction and qRT-PCR

Primer	Sequences (5′-3′)
ClbA- F	CCCATACTATAGATACAGTAGCCGTTGCTATTAGTTCTCACT GCGAGCTTGGTGTCGTGTGTAGGCTGGAGCTGCTTCG
ClbA - R	ACGCATAGCGCTCTCCTTCGTTGGCAGATTGATTATGCCGG ATGATCTCTCATATGAATATCCTCCTTAG
ClbA -VF	TGGCACAAGGTGATGAGTA
ClbA -VR	AGGTGGCTTCAGAGATTCC
pBRclbA-F	TAACGCAGTCAGGCACCGTGTGCTATACACATTGCTAACA GGAATGAG
pBRclbA-R	GTGAATCCGTTAGCGAGGTGCCTCAATTCTGCCCATTTGAC GAATG
TNF-α- F	ACTGAACTTCGGGGTGATCG
TNF-α- R	TGATCTGAGTGTGAGGGTCTGG
IL-1β- F	ATGAAAGACGGCACACCCAC
IL-1β- R	GCTTGTGCTCTGCTTGTGAG
IL-6- F	TGCAAGAGACTTCCATCCAGT
IL-6- R	GTGAAGTAGGGAAGGCCG
GAPDH-F	AACGGGAAGCCCATCACCATC
GAPDH-R	AAGACACCAGTAGACTCCACGA
