# Supplementary figures and images for: Phosphopantetheinyl transferase ClbA contributes to the virulence of avian pathogenic Escherichia coli in meningitis infection of mice
Source: PLoS One. 2022 Jul 28;17(7):e0269102. doi: 10.1371/journal.pone.0269102 (PMC9333332; doi:10.1371/journal.pone.0269102)

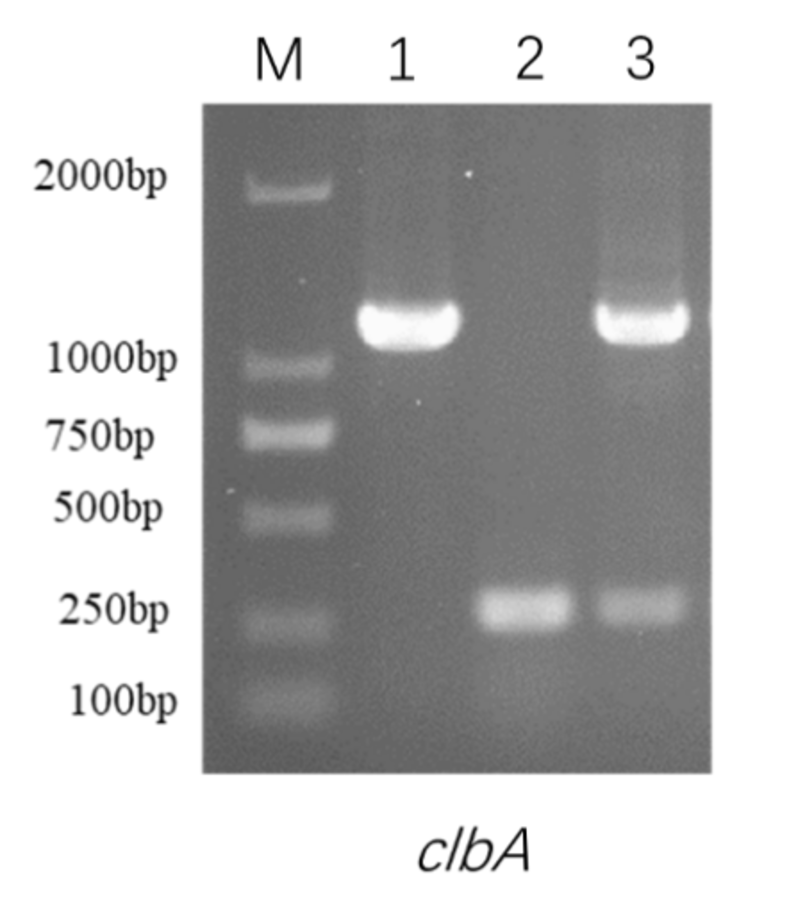

Supplement: S1 Fig — The mutants were identified by PCR amplification using primers ClbA -VF and ClbA-VR. About a 1230bp PCR product was obtained when the wild-type APEC XM genome as template (lane 1). APEC XMΔclbA mutant was verified by amplifying a 260bp fragment (lane 2). Both a 1230bp fragment and a 260bp fragment were obtained in APEC XMΔclbA/pclbA (lane 3). M: DL2000 DNA Marker. (TIF) [file pone.0269102.s002.tif]

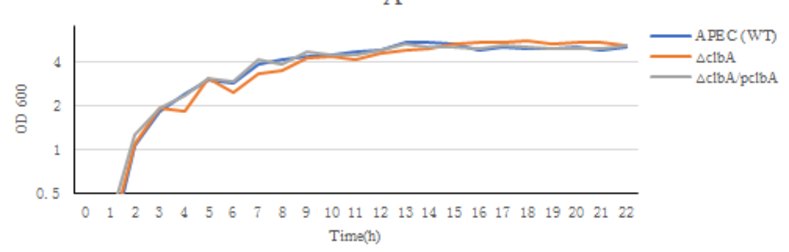

Supplement: S2 Fig — OD600 nm values of triplicate cultures in LB medium were determined at 1h intervals. Data are the means of three independent experiments. (TIF) [file pone.0269102.s003.tif]

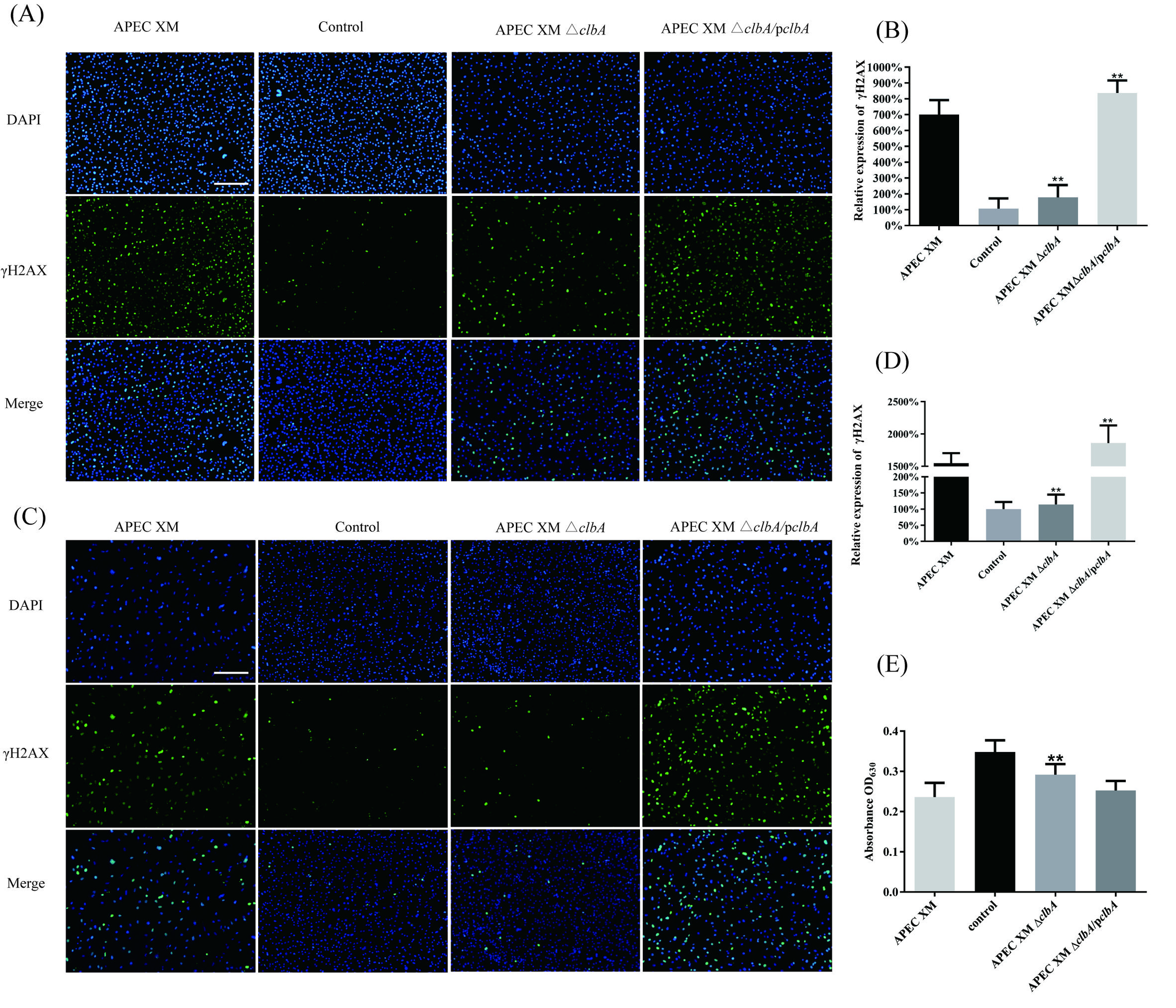

Supplement: S3 Fig — Immunofluorescence staining assay of γH2AX protein in bEnd.3 cells at 0 hpi (A) and 72 hpi (C). The nuclear DNA and γH2AX are colored blue and green, respectively (bar = 200 μm). Quantification of DNA double-strand breaks (DSBs) by calculating the percentages of γH2AX positive cells within the total cells at 0 hpi (B) and 72 hpi (D), normalized to the APEC XM infection group. The ratio of the APEC XM group was set as 100%. (E) Megalocytosis was quantified by determining the absorbance values at 630 nm. The results are given as mean ± standard error and analyzed with one‐way ANOVA (**, p < 0.01, versus APEC XM group). All assays were performed with three independent biological replicates. (TIF) [file pone.0269102.s004.tif]
