# Supplementary material for: Phosphopantetheinyl transferase ClbA contributes to the virulence of avian pathogenic Escherichia coli in meningitis infection of mice
Source: PLoS One. 2022 Jul 28;17(7):e0269102. doi: 10.1371/journal.pone.0269102 (PMC9333332; doi:10.1371/journal.pone.0269102)
Supplement: S1 Raw images — (PDF) [file pone.0269102.s005.pdf]

Original images of S1 Figure for gels

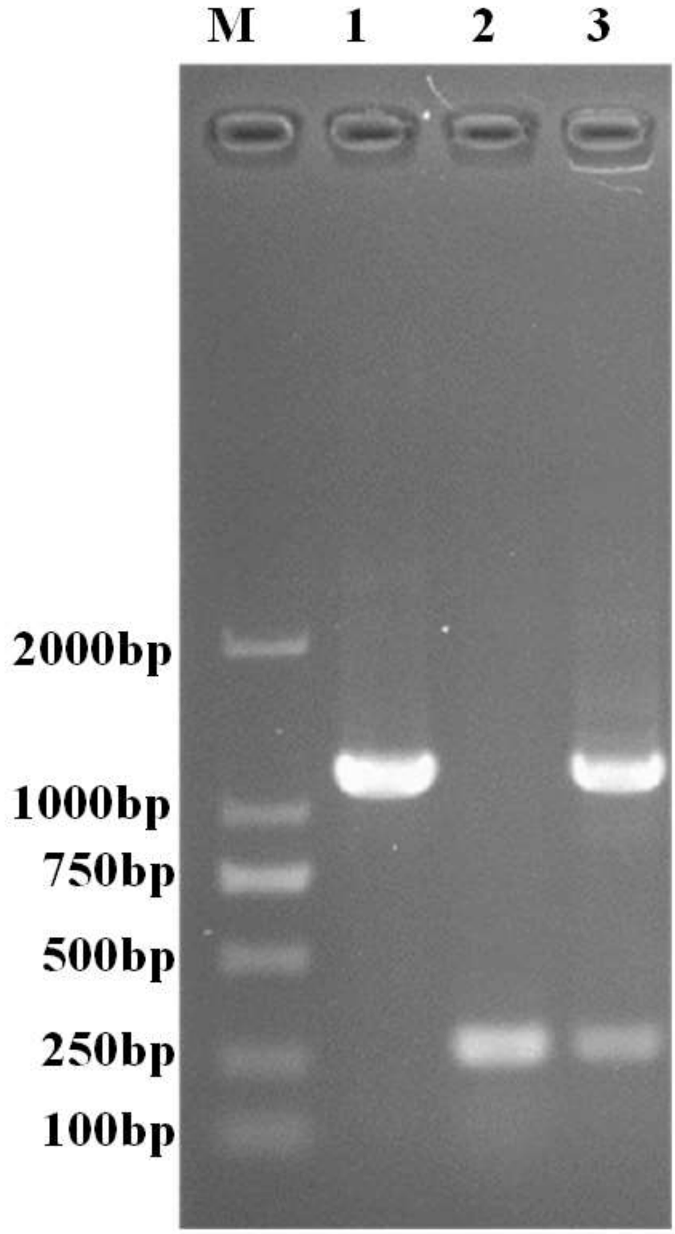

M: DL2000 DNA marker

1. APEC XM
2. APEC XMΔ*clbA*
3. APEC XMΔ*clbA*/p*clbA*
