# Supplementary material for: Phosphopantetheinyl transferase ClbA contributes to the virulence of avian pathogenic Escherichia coli in meningitis infection of mice
Source: PLoS One. 2022 Jul 28;17(7):e0269102. doi: 10.1371/journal.pone.0269102 (PMC9333332; doi:10.1371/journal.pone.0269102)
Supplement: S2 Raw images — (PDF) [file pone.0269102.s006.pdf]

Original images of Figure 3 for blots

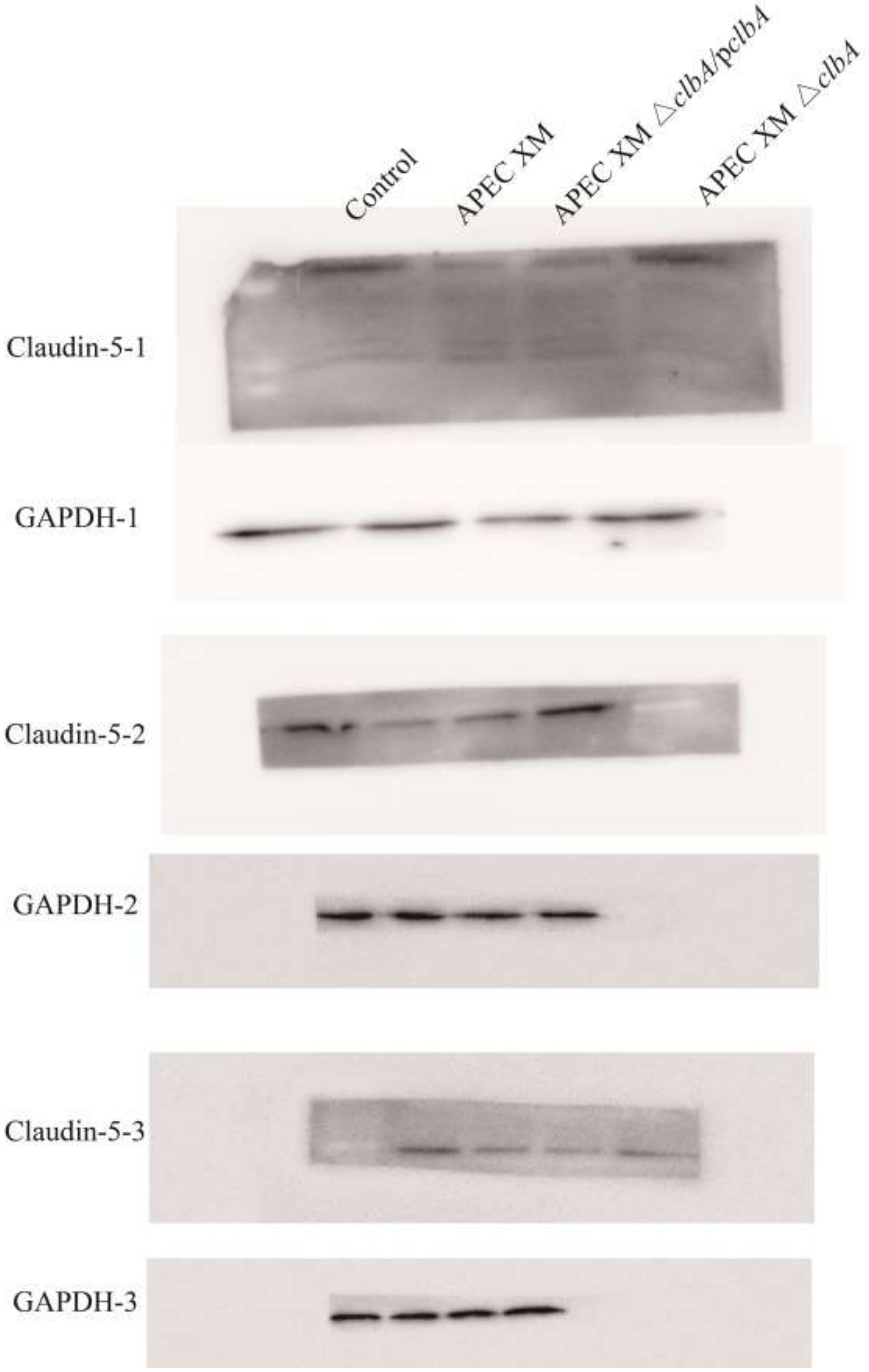

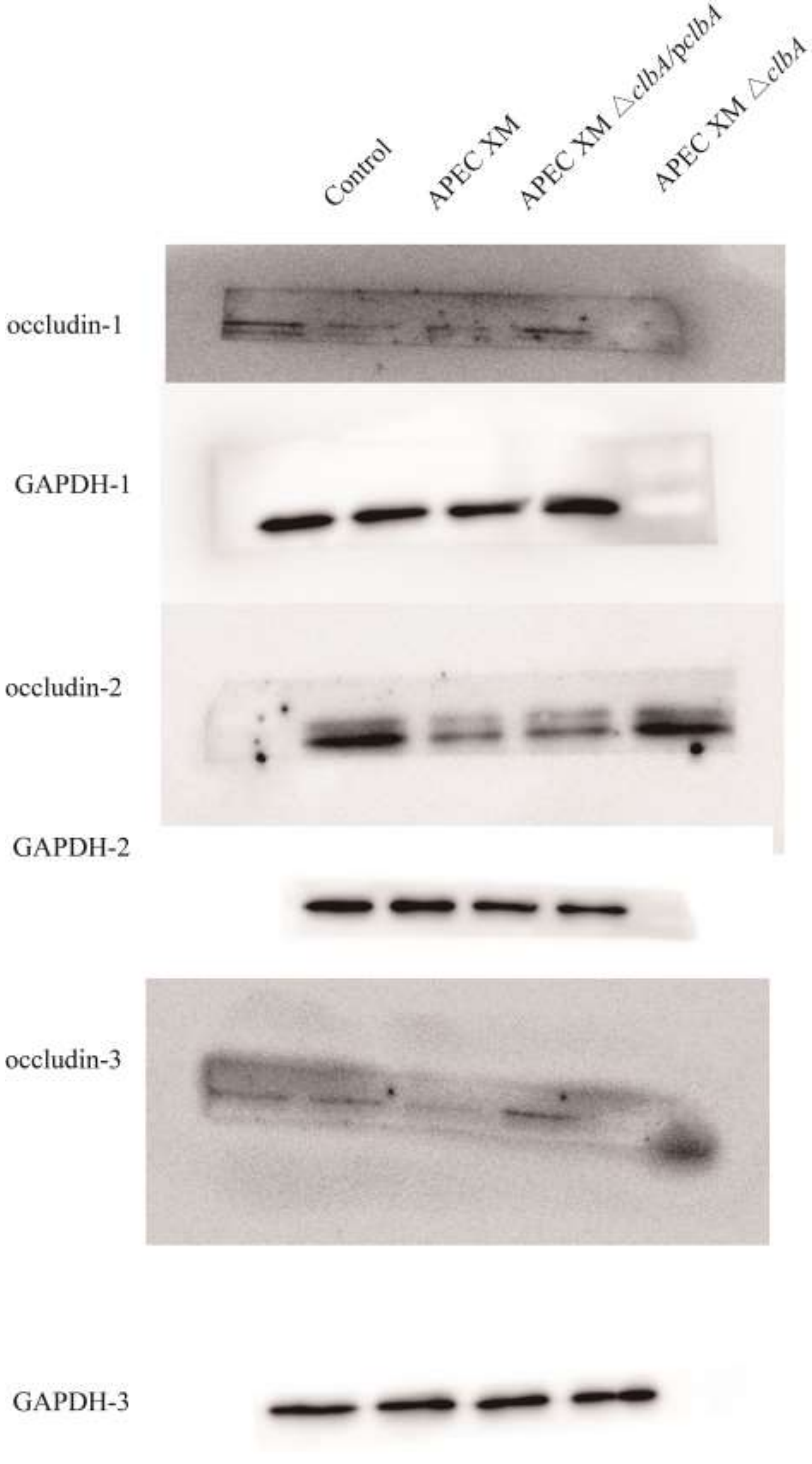

Control
APEC XM
APEC XM △clbA/pclbA
APEC XM △clbA
occludin-1
GAPDH-1
occludin-2
GAPDH-2
occludin-3
GAPDH-3

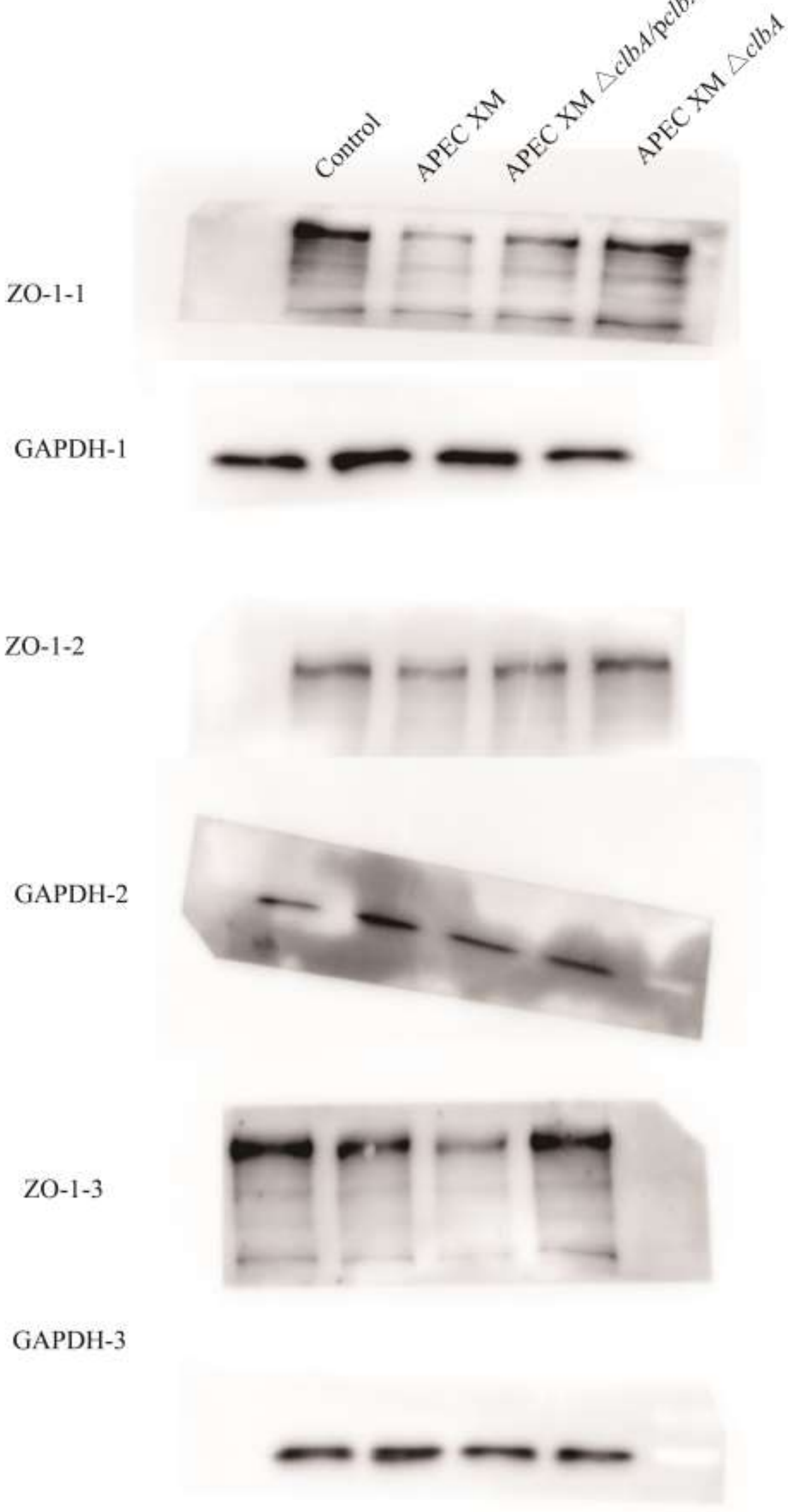

Control
APEC XM
APEC XM △clbA/pclbA
APEC XM △clbA
ZO-1-1
GAPDH-1
ZO-1-2
GAPDH-2
ZO-1-3
GAPDH-3
